# Designing and Evaluating IT Applications for Informal Caregivers: Protocol for a Scoping Review

**DOI:** 10.2196/47650

**Published:** 2023-07-06

**Authors:** Shweta Premanandan, Awais Ahmad, Åsa Cajander, Pär Ågerfalk, Lisette van Gemert-Pijnen

**Affiliations:** 1 Department of Informatics and Media Uppsala University Uppsala Sweden; 2 Division of Visual Information and Interaction Department of Information Technology Uppsala University Uppsala Sweden; 3 Centre for eHealth and Wellbeing Research Department of Psychology, Health & Technology University of Twente Enschede Netherlands

**Keywords:** burnout, caregiver, caregiving activity, design guidelines, design, development, digital health, digital resources, effectiveness, evaluation, health care services, implementation, informal caregiver, IT applications, long term care, mobile app, smartphone, usability, work-life balance

## Abstract

**Background:**

A growing number of informal caregivers in many countries are important for effective functioning of health care in our society. Hence, they must receive the required support and services to continue providing care. IT applications can be used to support informal caregivers in their caregiving activities. However, evidence-informed guidelines for developing such IT applications and their evaluation are scarce. Hence, this scoping review can aid researchers and designers by providing design recommendations for IT apps for caregivers and potentially improve the design of IT applications for caregivers to meet their needs better.

**Objective:**

This study aims to describe the proposal for a scoping review of current practices and recommendations in designing and evaluating IT applications for informal caregivers. The scoping review will also discuss the opportunities and challenges in designing these IT applications.

**Methods:**

We will use a 5-step scoping review methodology to map relevant literature published as follows: (1) identify the research question, (2) identify relevant studies, (3) select relevant studies for review, (4) chart the data from selected literature, and (5) summarize and report results. A structured search will be conducted in PubMed, Scopus, IEEE digital library, Web of Science, and ACM Digital Library databases. In addition, hand searches of reference lists and keyword searches in Google Scholar will also be conducted. Inclusion criteria will be researched (journal and conference) articles focused on IT applications designed for use by informal caregivers and study type to be qualitative studies. Two reviewers will independently identify articles for review and extract data. Conflicts will be discussed, and a third reviewer will be consulted if a consensus cannot be reached. These data will be analyzed using thematic analysis.

**Results:**

The results from this scoping review will be presented in a narrative form, and additional data on study characteristics will be presented in diagrams or tabular format. Uppsala University first initiated this scoping review protocol in December 2021 as part of the European Union–funded project ENTWINE. This work was also supported by the Swedish Research Council and the Swedish Cancer Society. The results will be presented in August 2023 and will be disseminated through a report to the European Union and a peer-reviewed journal publication. In addition, the team plans to share its findings on various public platforms, including social media, blog posts, and relevant conferences and workshops.

**Conclusions:**

This is, to our knowledge, the first study to map the literature on the design and evaluation of IT applications for informal caregivers. The scoping review’s results will detail the requirements, design suggestions, and user preferences, as well as the usability criteria and features of IT applications for informal caregivers. A mapping of studies could inform the design and implementation of future IT applications for informal caregivers.

**International Registered Report Identifier (IRRID):**

DERR1-10.2196/47650

## Introduction

### Overview

Informal caregivers voluntarily provide care for a relative or a friend facing illness, disability, or any condition requiring particular attention [1]. They are essential to the health care sector, providing critical care and support to those in need [2]. Informal caregivers play an important role in providing long-term care in people's homes and ensuring the quality of life for millions of individuals [3]. They must receive recognition and access to resources to help them better manage their responsibilities. Caregiving can be a burdensome role with negative consequences on the physical and emotional health of the caregiver [3]. Studies have consistently shown that informal caregiving is often associated with a sense of burden, resulting in psychiatric and physical illnesses and mortality [1,4,5]. Lack of choice in becoming a caregiver is often associated with a higher subjective burden [1,6]. Informal caregivers may experience burnout due to their role, which is characterized by feelings of exhaustion, lack of motivation, and difficulty concentrating [1]. This burnout can be caused by factors such as a lack of support from family and friends, financial strain, and feeling overwhelmed by the demands of caregiving [1]. The impact of informal caregiving on caregivers’ health has been studied extensively. Informal caregivers face several challenges, including limited personal time, difficulty navigating the health care system, and care recipients with a higher awareness of needs [7]. Organizations have introduced digital tools to address these challenges to support caregivers [8]. Additionally, recognizing and acknowledging their role is important for providing adequate support [8].

IT applications can benefit informal caregivers by assisting with their caregiving activities, among other things. IT applications for informal caregivers are technology-based tools that aim to provide support, resources, and assistance to individuals who provide care for family members or friends with chronic illnesses, disabilities, or aging-related issues. These applications can range from mobile apps to web-based platforms, for instance, the mobile app and web-based application CareHeroes [9]. They can provide educational information, help remember doctors’ appointments, and coordinate care among all involved parties [10]. Smartphone apps can deliver timely and personalized health information to families and friends of chronically ill adults living in the community [11]. Digital resources, such as webinars, handbooks, and more, can educate caregivers about conditions and improve care quality. IT applications can also support informal caregivers by streamlining the process of building an informal care team of friends and family members. By making it easier to share information, ask for help, and create sign-ups, tasks can be easily delegated from the informal caregiver to a wider network. Access to current information is essential for informal caregivers. They could benefit from technological services that provide up-to-date information on their loved one’s condition [12]. However, it is important to note that not all IT applications effectively support informal caregivers. A previous scoping review found that IT applications focus more on supporting caregivers in their caregiving role than their private and professional life [13]. Therefore, it is important to identify IT applications that cater to the primary caregiving and personal need domains of informal caregivers. This means developing IT applications that not only support caregivers in their caregiving roles but also address their personal and professional lives, such as managing their health, work-life balance, and social connections leading to better well-being. Overall, there is a need to investigate the design guidelines and principles for IT applications that go beyond addressing the caregiving role and cater to the personal and professional lives of informal caregivers, as well as to the specific needs of different caregiver groups. 

It is important also to evaluate the usability of IT applications for informal caregivers to ensure they are effective and useful. Research has shown that most applications offer limited evidence of effectiveness. This highlights the importance of conducting rigorous usability evaluations of IT applications to ensure that they are meeting the needs of their users and achieving their intended goals. Still, those evaluated have been found to help provide educational information, remember doctors' appointments, coordinate care among all caregivers, manage behavior problem checklists, provide alerts for care providers, and offer information about available services [10,14]. Evaluating IT applications for informal caregivers is important in identifying and addressing usability issues and ensuring that they are effective and useful for their intended purpose [14]. Caroço et al [15] and Gomes et al [16] report on the usability studies of Help2Care, an eHealth platform that provides digital training materials for informal caregivers. Both studies found that the platform had less usable aspects, such as navigation flow and design elements, which were identified through usability tests. Držanič et al [17] evaluated the usability of a smartphone telecare app for informal caregivers and found that users perceived the app as high in usability and user experience. Xiao et al [18] designed and evaluated web interfaces for informal caregivers in senior monitoring and found that combining evaluation methods is important in identifying usability issues. Overall, these papers suggest that usability evaluations are important in identifying and addressing usability issues in IT applications for informal caregivers.

There are some reviews in the field of IT applications for informal caregivers that have been discussed in recent literature. Sala-González et al [10] conducted a systematic review of mobile apps for helping informal caregivers, finding that most of the studies assessed caregivers’ needs before designing mobile apps to adapt them to the needs of their users which has proved useful. Martínez-Alcalá et al [19] and Powell et al [20] found that IT applications were useful in improving the life of informal caregivers. However, it was found that there were some challenges to deploy these IT solutions for informal caregivers, and Hassan [21] conducted a scoping review and provided recommendations for the deployment of IT applications for informal caregivers. Guessi Margarido et al [11] investigated the nature and extent of native smartphone apps for informal caregivers of chronically ill patients and found that most apps targeted a single chronic condition, with Alzheimer and other dementia being the most common. Meyer et al [22] conducted a systematic review of novel technology as a platform for interventions for caregivers and individuals with severe mental health illnesses and found that none of the studies focused on caregivers, and the ones identified using mobile or web-based apps were just for patients and not their relatives. While previous reviews have explored the effectiveness of IT applications and the challenges and recommendations for their deployment, our proposed scoping review aims to contribute to the existing literature on IT applications for informal caregivers by identifying and summarizing knowledge about the design recommendations and usability of such applications.

### Study Objectives

The objective of the proposed scoping review is to identify and summarize knowledge about the design recommendations of IT applications and the usability of IT applications by informal caregivers. The scoping review will also discuss the opportunities and challenges in designing these IT applications. Design recommendations for IT applications refer to best practices and guidelines that can be followed by software designers and developers to ensure that the applications they create are user-friendly, efficient, and effective. For instance, a relevant design recommendation could be accessible information; the application should provide concise and relevant information that is easily accessible and available in different formats, such as text, audio, and video, to cater to the different learning styles and needs of informal caregivers. 

Usability refers to the degree to which an IT application is easy to use, learn, and understand. A usable IT application is designed with the end user in mind, allowing users to complete their tasks quickly and efficiently without encountering unnecessary obstacles or confusion. Many IT applications are designed for caregivers based on identified unmet needs through interviews and focus groups. However, to the best of our knowledge, this literature has not been compiled. This scoping review can aid researchers and designers by providing design recommendations for IT applications for caregivers and potentially improve the design of IT applications for caregivers to meet their needs better.

## Methods

### Study Design

Arksey and O’Malley’s [23] methodological framework for scoping reviews will be used for the proposed scoping review. The research topic addressed here includes a wide range of study designs and various support platforms and solutions for informal caregivers caring for a wide range of illnesses and care-recipient types carried out in multiple countries. Moreover, designing effective solutions for informal caregivers has been carried out in multiple fields like information systems, human-computer interaction, and health informatics. A scoping review methodology was considered suitable as this is an emerging topic where evidence is scarce and scattered. There is a need to bring results from these different fields of designing IT support together to generate a common knowledge pool, which is a major research gap. This scoping review methodological framework outlines the following five stages: (1) identification of the research question; (2) identification of relevant studies; (3) selection of relevant studies; (4) charting the data from the selected literature; and (5) collating, summarizing, and reporting the results.

We will follow the PRISMA-ScR (Preferred Reporting Items for Systematic Reviews and Meta-Analysis Extension for Scoping Reviews) guidelines [24] to complement the conduct and reporting of the results.

### Step 1: Identifying the Research Questions

The focus of the proposed review was identified through a preliminary scan of the extant literature. Initially, the focus was the design recommendations for IT applications for informal caregivers. However, upon initial inquiry, it became clear that to have effective and user-friendly design recommendations, the review would benefit from also looking at the evaluation of IT applications for informal caregivers. The research question was also discussed, and feedback was obtained from experts from Uppsala University and the eHealth research group at the University of Twente. Based on multiple rounds of brainstorming, the following questions were developed for the scoping review:

1. What are the design recommendations for developing IT applications for informal caregivers?
2. How are these IT applications perceived and evaluated by informal caregivers?

### Step 2: Identification of Relevant Studies

The literature search was conducted in collaboration with a health psychology information specialist at the University of Twente and some sessions with the university librarian at Uppsala University. Search terms focused on three components of the research question: (1) informal caregivers, (2) IT applications, and (3) design or evaluation. Terms related to caregivers were based on previous reviews on this topic from Lambert et al [25,26] and included terms, such as “home nursing,” “informal caregiver,” “family caregiver,” “informal carer,” “family carer,” “caregiver,” and “carer.” Terms related to IT solutions included “mobile application,” “ICT solution,” “ICT,” “e-coaching system,” “coaching system,” “digital solution,” and “IT solution.” Terms related to design and evaluation included “design,” “evaluation,” “effectiveness,” “usability,” “requirements,” “needs,” “perspective,” and “user experience.” Table 1 provides an overview of the different groups of keywords.

Studies included both conference papers and journal articles as publication types. The databases searched were PubMed, Scopus, IEEE digital library, Web of Science, and ACM Digital Library. EndNote 12 was used to store the search results and remove duplicates. The quality of the searches was confirmed by verifying that the results contained relevant studies identified before the formal search.

**Table 1:** Keywords for the search string.

Keywords	Search terms
Group 1: informal caregiver	“Home nursing” OR “informal care*” OR “family care*”
Group 2: IT solution	“Mobile application*” OR “ICT solution*” OR “ICT” OR eHealth OR “e-coaching system*” OR “* coaching system*” OR “digital solution*” OR “IT solution*” OR “internet-based interventions*” OR telehealth*
Group 3: design or evaluation	Design OR evaluation OR effectiveness OR usability OR requirements OR needs OR perspective OR “user experience*”

### Step 3: Selection of Relevant Studies for the Review

**Figure 1:**
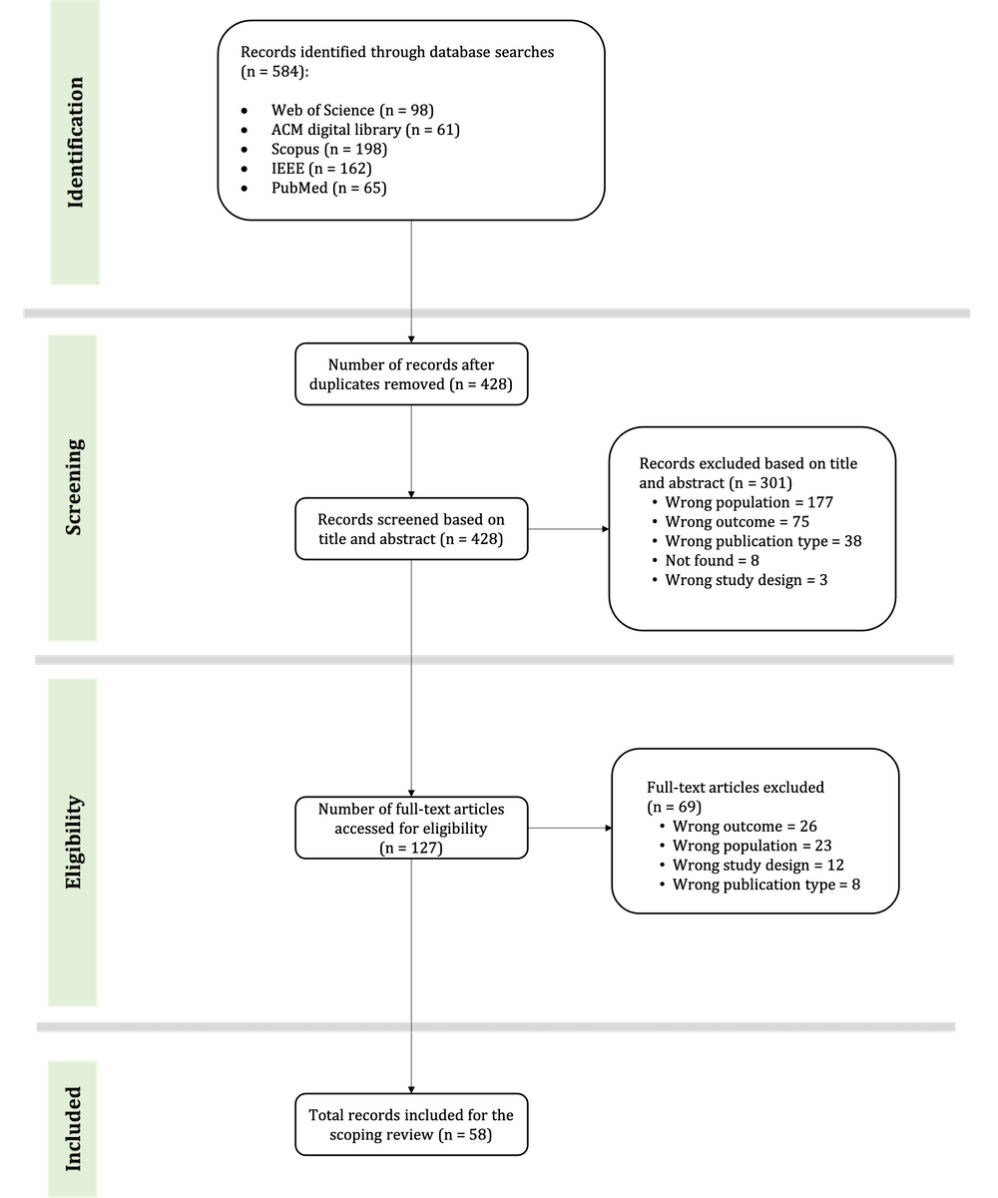
Study selection flow diagram.

The initial screening of titles and abstracts was done only by the first author (SP) and was guided by the inclusion criteria using the web-based screening tool Rayyan [27]. At present, SP has also completed the full-text screening stage, while the second author (AA) will soon start full-text screening of all the articles. In the event of conflicts, a discussion will be held between the authors, and a third reviewer will be consulted if a consensus cannot be reached. Studies will be included if they are peer-reviewed conference articles, journal articles, or early-access articles. As the scoping review is restricted to peer-reviewed articles, the next stage includes removing proceedings, editorial and opinion papers, news articles, books and book chapters, theses, and clinical case papers. The inclusion and exclusion criteria to be used are summarized in Textbox 1. The study selection process is illustrated in Figure 1. The number of articles in each stage indicates the numbers from the first review. These numbers are expected to change based on the second review and further discussions.

**Inclusion and exclusion criteria.**

**Inclusion criteria:**

- Publication type: conference articles, journal articles, and early access articles
- Studies regarding usability tests of IT apps or platforms
- Studies describing user needs or requirements for IT apps for informal caregiving
- Studies that describe the evaluation of IT apps focusing on what works or what is preferred along with user experience
- Studies describing the development process of IT apps for informal caregiving
- Studies on IT apps to train informal caregivers in their caregiving activities

**Exclusion criteria:**

- Study type: review articles, editorial and opinion papers, news articles, books and book chapters, theses, and clinical case papers
- Descriptive or quantitative studies assessing the adoption of a system or criteria for adoption based on a theoretical model
- Studies focused on care recipients and formal caregivers
- Studies on IT apps targeted at patients, but developed with interviews with patients and informal caregivers along with health care professionals

### Step 4: Charting the Data

The following information regarding the included studies will be extracted and stored in Excel (Microsoft Corp): authors, year of publication, the purpose of the paper or study and innovation, study location and context (setting and end users of innovation), study design, outcomes measured, main findings, and lessons learned used. Once the analysis and writing of the scoping review manuscript are finished, the articles used for the review will be accessible on the open science framework platform [28]. While submitting the scoping review manuscript, full citations of the included articles will also be added as supplementary files.

### Step 5: Collating, Summarizing, and Reporting the Results

A descriptive-analytical narrative method was used to extract and chart the data from the selected articles. Using the same process of team consultation, data from the selected articles were first extracted onto a data charting form developed by the research team using an iterative process. Charts were used to collate, summarize, and share data for team review and decision-making. Data entered included the following: authors, year of publication, the purpose of the paper or study and innovation, study location and context (setting and end users of innovation), study design, outcomes measured, main findings, and lessons learned. A coding scheme (framework) will be created under three thematic categories: (1) design and development, (2) evaluation, and (3) opportunities and challenges. Full articles were imported as PDF files into NVivo 12 (QSR International), a software program for qualitative analysis, for more detailed data extraction and coding. The authors applied the coding scheme to all pertinent text, and then further coded the data under emergent themes using an iterative process.

## Results

The findings of our scoping review will be presented in a clear and coherent narrative, supplemented by relevant data on the study characteristics in diagrams or tables. The results can be broadly categorized into 2 main sections: design recommendations and usability criteria. Under the design recommendations category, we may have subcategories that address specific conditions or that are based on the type of system or caregiver characteristics. This will provide a more tailored approach to designing eHealth applications for informal caregivers that take into account their unique needs and circumstances. Similarly, the usability criteria category may also have subcategories that are specific to certain types of systems or caregivers. By considering the context in which caregivers use these applications, we can identify usability criteria that are most important to their overall experience. The presentation of our results will provide valuable insights for researchers and designers working in the field of eHealth applications for informal caregivers. The subcategories will help to further refine our understanding of the needs and preferences of this population and guide the development of more effective and user-friendly applications. The scoping review will also discuss the opportunities and challenges in designing these IT applications.

Uppsala University first initiated this scoping review protocol in December 2021 as part of the European Union–funded project ENTWINE. This work was also supported by the Swedish Research Council and the Swedish Cancer Society. The scoping review is being undertaken to delve into the potential design recommendations and usability criteria for IT applications catering to informal caregivers. The main objective of this scoping review is to produce 2 comprehensive lists: one comprising design recommendations and the other focusing on usability criteria for IT applications targeted toward informal caregivers. The first list will serve as a solid foundation for designers who are keen to develop IT applications for informal caregivers and will provide the answers to the first research question. If the chosen articles address both design recommendations and usability criteria for IT applications, the team will be able to effectively gauge whether these design recommendations are well-received by the caregivers. However, if some included papers only discuss usability criteria, the resulting list would still serve as a starting point for designers, as it would provide a comprehensive set of usability criteria for designing IT applications for caregivers and also help answer the second research question.

The results of the scoping review will be presented in August 2023 and will be disseminated through a report to the European Union and a peer-reviewed journal publication. In addition, the team plans to share its findings on various public platforms, including social media, blog posts, and relevant conferences and workshops.

## Discussion

In this proposed scoping review, we will provide an overview of the current state of research on the design and usability evaluations of IT applications for informal caregivers. The review will identify the types of IT applications, their design recommendations, and the usability criteria. This information can guide the development of new IT applications or improve existing ones. The scoping review will also provide an opportunity to reflect on the challenges and opportunities associated with designing and evaluating IT applications for informal caregivers. To our knowledge, no previous studies have explicitly highlighted these factors. While Hassan’s [21] study addressed the challenges and recommendations for the practical implementation of IT applications for informal caregivers, it solely focused on the caregivers of older adults. On the other hand, our study seeks to broaden the scope and delve into the factors essential for designing and evaluating IT applications for all informal caregivers.

The potential benefits of IT applications for informal caregivers are numerous [29,30]. They can provide support tailored to the individual caregiver’s needs [2,31]. For example, an IT application could provide personalized reminders to take breaks or offer suggestions for self-care activities. IT applications can also connect caregivers to support networks, such as other caregivers or health care professionals [32,33]. This can help reduce the social isolation that is often associated with being an informal caregiver. Such apps can provide support for caregivers in the form of information, education, communication, and coordination of care. They also offer solutions for specific caregiver challenges, such as medication management and self-care. Moreover, these apps can potentially reduce caregiver burden, stress, and burnout, thereby improving the quality of care provided to the care recipients. 

However, designing and evaluating IT apps for informal caregivers is a complex and challenging task. Many factors must be taken into account, such as the needs and preferences of caregivers, the technological capabilities of the target population, and the ethical and privacy considerations involved [21,34,35]. Additionally, evaluating the effectiveness of IT applications for informal caregivers requires using appropriate iterative evaluation methods that consider the unique characteristics of this population [29,36].

Our scoping review thus provides a comprehensive overview of the unique challenges and opportunities associated with designing and evaluating the usability of IT applications for informal caregivers across all age groups. The findings of our study will help researchers and developers create more effective and efficient eHealth interventions that cater to the diverse needs of informal caregivers.

## Abbreviations

PRISMA-ScR: Preferred Reporting Items for Systematic Reviews and Meta-Analysis Extension for Scoping Reviews

## Data Availability

Once the analysis and writing of the scoping review manuscript are finished, the articles used for the review will be accessible on the open science framework platform. While submitting the scoping review manuscript, full citations of the included articles will also be added as a supplementary file.
